# Le traumatisme du colon: l'expérience du CHU Hassan II de Fès

**Published:** 2012-11-21

**Authors:** El Bachir Benjelloun, Hasnai Hafid, Ibnmajdoub Karim, Abdelmalek Ousadden, Khalid Mazaz, Kahlid Ait Taleb

**Affiliations:** 1 Department of surgery, University hospital Hassan II, Fez, Morocco

**Keywords:** Traumatisme du colon, réparation primaire, colostomie, Colon trauma, primary repair, colostomy

## Abstract

**Introduction:**

Les traumatismes du colon sont associés à un risque majeur de complications septiques et de mortalité. Le but de notre étude est d’évaluer les circonstances, la prise en charge, le suivi et les facteurs pronostic de morbidité postopératoire des malades victimes d'un traumatisme colique.

**Méthodes:**

Il s'agit d'une étude rétrospective sur une série de 49 patients opérés pour des plaies coliques aux services de chirurgie viscérale du CHU HASSAN II de Fès sur une période de 8 ans de juillet 2003 à juillet 2011.

**Résultats:**

L’âge moyen de nos patients était de 25ans (16-70) avec une nette prédominance masculine (93.8%). Les plaies coliques secondaires à un traumatisme par arme blanche représentent 85% des cas (42 patients), suivi par les plaies iatrogènes au cours d'une coloscopie chez 6 patients (13%), puis les contusions abdominales chez 1 patient (2%). Les parties du cadre colique les plus touchées étaient le colon transverse chez 19 patients (38%) et le colon descendant chez 12 patients (24, 5%). Le colon sigmoïde était le segment le plus touché au cours d'une coloscopie4/6. Quarante-deux patients (85%) ont eu une suture primaire des plaies coliques, six patients (13%) une diversion fécale et un patient (2%) une résection-anastomose. Deux patients (4%) sont décédés suite à un choc septique. La morbidité globale était de 38,7% dominé essentiellement par l'infection de la paroi chez 14 patients et une péritonite post opératoire chez 3 patients. L'analyse univarié a montré une différence significatif en terme d'infection de la paroi entre le groupe colostomie versus suture simple (50% vs 20,9% p<0,05). L'atteinte du colon gauche et la réalisation d'une colostomie sont associés à un risque plus élevés de complications postopératoires.

**Conclusion:**

La suture primaire peut être effectuée avec un faible taux de complications postopératoire chez la majorité des patients suite à un traumatisme du colon.

## Introduction

La philosophie de la prise en charge des traumatismes du côlon est en constante évolution. Le réflexe de la création d′une colostomie en réponse à une contamination fécale est remis en question avec l'apparition de plusieurs travaux appuyant la réparation colique première [1]. Cependant, la gestion des traumatismes étendus du côlon par résection et anastomose immédiate reste controversée [2]. Récemment, une étude prospective, randomisée et multicentrique, a montré que le type de traitement chirurgicale n'a pas d'influence sur les complications septiques [3]. A Fès le traumatisme du colon par armes blanches est le plus fréquent contrairement à d'autres régions du monde ou le traumatisme colique est surtout dû aux armes à feu et aux traumatismes fermés de l'abdomen. Ça peut être explique par l’âge jeune de la population et sa concentration dans des quartiers avec un bas niveau socioéconomiques.

Dans ce travail nous présentons 8 ans d'expérience du CHU Hassan II de Fès dans la prise en charge des traumatismes coliques, en essayant de définir les facteurs de risque des complications septiques et les circonstances dans lesquelles la réparation première est non seulement indiquée, mais aussi supérieure à la diversion fécale.

## Méthodes

Il s'agit d'une étude rétrospective d'une série de 49 patients opérés pour des plaies coliques au service de chirurgie viscérale du CHU HASSAN II de Fès sur une période s’étalant sur 8ans de juillet 2003 à juillet 2011. Etaient inclus tous les patients opérés pour une plaie colique avec ou sans autres lésions associées suite à un traumatisme abdominal ou après une coloscopie. Les traumatismes anorectaux associés à un traumatisme colique ont été exclus de l’étude. Les dossiers des patients ont servi de base pour le recueil des données.

Les plaies coliques ont été gérées soit par une réparation primaire, résection avec anastomose, ou diversion fécale (colostomie). La décision peropératoire était fondée sur la gravité des blessures du côlon, le degré de contamination fécale, la localisation anatomique de la blessure, le délai entre le traumatisme et l'admission et la présence d'autres lésions associés. Les variables étudiés sont: l’âge des patients, le sexe, le mécanisme du traumatisme, le délai de consultation, la localisation anatomique de la blessure, la présence d'un état de choc à l'admission, les lésions abdominales associés, le type de réparation colique et les complications postopératoires.

Ces variables ont été analysées par rapport au développement de l′infection du site opératoire et de complications intra-péritonéales afin d′identifier les potentiels facteurs de risque de morbidité postopératoire. Student′s t-test et chi-squared test ont été utilisé pour l'analyse statistique.

## Résultats

Les caractéristiques générales des 49 patients analysés inclus un âge moyen de 25 ans (intervalle: 16 à 70 ans), dont 93,8% sont de sexe masculin. Les plaies coliques secondaires à un traumatisme par arme blanche représentent 85% des cas, suivi par les plaies iatrogènes au cours d'une coloscopie chez 6 patients (13%), puis les contusions abdominales chez 1 patient (2%). Le délai entre le traumatisme et la chirurgie était moins de 6h chez 81,6% des patients. Le flanc gauche constitue la région topographique la plus touchée par le traumatisme (24%). Le tableau clinique était dominé par une défense abdominale chez la majorité des patients. La radiographie pulmonaire et l’échographie abdominale étaient les principaux examens paracliniques réalisés. Le pneumopéritoine n'a été retrouvé que chez 10,2% des patients. L’échographie abdominale a objectivé un épanchement intrapéritonéale chez 34,6% des cas. L'hyperleucocytose a été notée dans 51% des cas. Les parties du cadre colique les plus touchées étaient: le colon transverse (32%), le colon sigmoïde (20%), le colon descendant (20%) et le colon ascendant dans 10% des cas (Tableau 1). Le colon sigmoïde était la partie la plus touché suite aux perforations iatrogènes au cours d'une coloscopie 4/6. Les lésions associées étaient dominées par les plaies du grêle retrouvé dans 30,6% des cas suivi par les plaies de l'estomac dans 10,2% des cas (Tableau 1). Quatre patients se sont présentés en état de choc (définie comme une pression systolique pression artérielle <90 mm Hg) à l'admission. L'indication opératoire a été posé d'emblée chez 47 patients soit (85,7% des cas) tandis que la décision de surveillance clinique et biologique avait été décidé chez 2 patients (4%) opérés ultérieurement pour apparition de défense abdominale avec hyperthermie. L'exploration chirurgicale a trouvé un épanchement intra péritonéal hémorragique chez 55,1% des cas, stercoral chez 2 patients (8% des cas). Les gestes réalisés étaient: une réparation primaire par suture simple chez 44 patients (89,7%), une colostomie chez 5 patients (10,3%) et une résection-anastomose chez un seul patient (2%). La morbidité globale était de 38,7% dominé essentiellement par l'infection de la paroi chez 14 patients, une péritonite post opératoire chez 3 patients et un abcès peristomial chez 1 patient (Tableau 2). La mortalité post opératoire était de l'ordre de 4% (2 cas), suite à un choc septique.

**Tableau 1:** Caractéristiques cliniques des patients et lésions abdominales associées

**Données patients**	
Age moyen (années)	25
Extrêmes d’âges	(25-70)
Sexe ratio (Homes/Femmes)	4/1
**Type de traumatisme**	
Plaie pénétrante par arme blanche	42
Traumatisme fermée de l'abdomen	1
Plaie iatrogène au cours d'une coloscopie	6
**Localisation des plaies coliques**	
Colon ascendant	
Colon transverse	
Colon descendant	
Colon sigmoïde	
**lésions abdominales associées (n=29)**	
Grêle	15 (51.7%)
Estomac	5 (17.2%)
Rate	2 (6.8%)
Foie	2 (6.8%)
Mésentère	3 (10.3%)
Diaphragme	1 (3.4%)
Vésicule biliaire	1 (3.4%)

**Tableau 2:** Complications postopératoires

Type de complications	Totale (n=19)
Fistule stercorale	1 (5.3%)
Infection de la paroi	14 (73.7%)
Abcès peristomiale	1 (5.3%)
Péritonite postopératoire	3 (15.7%)

L'analyse Univarié des facteurs pronostiques de morbidité postopératoire a révélé comme facteurs de risques parmi les variables étudiés: le traumatisme du colon gauche et la réalisation d'une colostomie (Tableau 3). La morbidité et la mortalité associées aux traumatismes du côlon ont été évaluées par rapport aux méthodes de réparation (Tableau 4). Les complications infectieuses superficielles (infection de la paroi, abcès peristomial) sont plus fréquentes dans le groupe colostomie versus suture simple (50% vs 20,9% p0,05).

**Tableau 3:** Les facteurs de risque de complications postopératoires

Facteurs pronostic	Taux de complications	P value
**Délai moyen entre traumatisme et chirurgie**		
< 6h	12/40(30%)	> 0.05
>6h	2/9 (22.2%)	
**Lésions abdominales associés**		
Oui	7/29 (24.1%)	> 0.05
Non	7/20 (35%)	
**Mécanisme du traumatisme**		
Plaie pénétrante par arme blanche	11/42 (26.1%)	> 0.05
Coloscopie	2/6 (33.3%)	
**Localisation colique**		
Colon droit	3/21 (14.2%)	< 0.05
Colon gauche	11/28 (39.2%)	
Suture simple	11/43 (25.5%)	< 0.05
Colostomie	3/6 (50%)	

**Tableau 4:** Morbidité et mortalité associé au type de la chirurgie

Morbidité/Mortalité	Groupe suture primaire	Groupe colostomie	P value
Complications infectieuse superficielles	9/43 (20.9%)	3/6 (50%)	< 0.05
Complications infectieuse profondes	2/43 (4.6%)	0/6 (0%)	> 0.05
Décès	2/43 (4.6%)	0/6 (0%)	> 0.05

## Discussion

L′évolution de la prise en charge du traumatisme du colon a connu un changement majeur ces deux dernières décennies. Durant la première guerre mondiale, la réparation primaire était préférée dans le traitement des plaies du côlon, avec un taux de mortalité allant jusqu'a 75% [4, 5]. En 1943, pendant la seconde guerre mondiale, les autorités médicales militaires des Etats-Unis ont exigé la réalisation d'une colostomie pour toute plaie colique, indépendamment du mécanisme du traumatisme [6]. Ces recommandations ont conduit à une importante diminution de la mortalité 30% à 35% [7, 8]. Jusqu'au trois dernières décennies la diversion fécale est devenu un standard dans les traumatismes du colon. Une grande partie de notre approche actuelle dans les traumatismes coliques est basé sur le travail de Stone et Fabian. En 1979 ils publient la première étude prospective randomisée comparant la réparation primaire à la diversion fécale dans la prise en charge des plaies coliques dans les traumatismes civils [9]. Ils ont conclu que la réparation primaire était meilleure que la colostomie dans des cas sélectionnés. Depuis, plusieurs études prospectives et rétrospectives ont suivi soutenant cette démarche, tout en insistant sur les critères de sélection des patients. Shannon et Moore ont définis ces critères par une stabilité hémodynamique du patient avec un indice de traumatisme abdominal <25 [10]. Shultz et al ont ajouté l'absence de traumatisme intra abdominale associé ainsi qu'un faible score de Flint (score des traumatismes du colon)[11]. Les critères décrits par Adkins et al incluent l′intervalle de temps entre la blessure et la chirurgie ainsi que l′état général du patient [12].

Dans notre institution, la réparation primaire est devenue la procédure de choix dans les traumatismes du colon. Pour les patients à haut risque (admission en état de choc, contamination abdominale diffuse, retard d'admission) nous préférons utiliser une diversion fécale comme première étape du traitement. Dans notre série, nous étions en mesure de comparer les résultats entre les différentes approches chirurgicales (réparation primaire vs diversion fécale). Le taux de complication pour la cohorte entière était de (38,7%). Les caractéristiques des patients, la morbidité, et le taux de mortalité n′étaient pas significativement différents lorsque nous avons comparé le groupe (suture primaire versus colostomie). Les complications infectieuses superficielles (infection de la paroi, abcès peristomiale) sont plus fréquentes dans le groupe colostomie versus suture simple (50% vs 20,9%), tandis que les complications infectieuses profondes sont plus fréquentes dans le groupe suture simple (4,6% vs 0%) mais sans être significatif. Dans l’étude de George et al, [13] les facteurs de risque pour les complications septiques indépendamment du type de la chirurgie (suture primaire ou colostomie) incluent la nécessité de transfusion sanguine de plus de 4 Culot Globulaire, l'existence de plus de 2 plaies coliques, une contamination péritonéale importante et un haut indice de traumatisme abdominal. Dans notre série, l'analyse des facteurs de risque des complications septiques postopératoire a révélé le traumatisme du colon gauche et la réalisation d'une colostomie comme facteurs de risques parmi les variables étudiés. Paradoxalement d'autres facteurs comme: un délai moyen > 6 heures entre le traumatisme et la chirurgie, l'association d'autres lésions intrapéritonéales (grêle, estomac..) ou le mécanisme du traumatisme (plaie pénétrante par arme blanche ou plaie iatrogène au cours d'une coloscopie) ne contribue pas à une augmentation de complications postopératoires (Tableau 3). Plusieurs études ont montré qu'un délai plus de 12 heures, avec même une contamination fécale ne présente pas une contre-indication à la suture primaire [14–17].

Une revue de la littérature récente de près de 3.000 cas de lésions coliques dans une population civile a rapporté un taux de lâchage de 5,5% après résection-anastomose et seulement 1,4% après réparation primaire [18]. Dans notre série, il y avait 2 cas de lâchage dans le groupe suture primaire 2/43 (4,6%). Le principal avantage de la réparation primaire est d’éviter la morbidité associée à la colostomie et sa fermeture. Sur la base des données de la littérature, le taux de complications rapporté pour la fermeture de la colostomie est entre 20% et 55% [19, 20] avec un délai moyen de rétablissement de la continuité de 2 à 6 mois. Chez nos patients la fermeture de colostomie a été réalisée dans un délai minimum de 2 mois. Aucun cas de lâchage anastomotique ou de fistule stercorale n'a été signalé, cependant l'infection de la paroi a été notée chez 70% des patients.

## Conclusion

Notre étude montre que notre prise en charge et nos résultats sont proches à ceux de la littérature. La suture primaire est la méthode de choix dans les traumatismes du colon. Elle est efficace, moins couteuse avec un faible taux de complications septiques. Cependant les indications traditionnelles de la colostomie (péritonite stercorale, choc septique, chirurgie destructive majeur) demeurent toujours valables de nos jours pour une prise en charge plus sécurisée.
